# Ethics, Risk and Benefits Associated with Different Applications of Nanotechnology: a Comparison of Expert and Consumer Perceptions of Drivers of Societal Acceptance

**DOI:** 10.1007/s11569-015-0222-5

**Published:** 2015-04-24

**Authors:** N. Gupta, A. R. H. Fischer, L. J. Frewer

**Affiliations:** 1 Marketing and Consumer Behaviour Group, Wageningen University, 6706 KN Wageningen, The Netherlands; 2 Food and Society Group, School of Agriculture, Food and Rural Development, Newcastle University, Newcastle Upon Tyne, NE1 7RU UK

**Keywords:** Ethical concern, Expert-lay comparison, Nanotechnology, Consumer perception, Repertory grid method, Societal acceptance

## Abstract

Examining those risk and benefit perceptions utilised in the formation of attitudes and opinions about emerging technologies such as nanotechnology can be useful for both industry and policy makers involved in their development, implementation and regulation. A broad range of different socio-psychological and affective factors may influence consumer responses to different applications of nanotechnology, including ethical concerns. A useful approach to identifying relevant consumer concerns and innovation priorities is to develop predictive constructs which can be used to differentiate applications of nanotechnology in a way which is meaningful to consumers. This requires elicitation of attitudinal constructs from consumers, rather than measuring attitudes assumed to be important by the researcher. Psychological factors influencing societal responses to 15 applications of nanotechnology drawn from different application areas (e.g. medicine, agriculture and environment, food, military, sports, and cosmetics) were identified using repertory grid method in conjunction with generalised Procrustes analysis. The results suggested that people differentiate nanotechnology applications based on the extent to which they perceive them to be beneficial, useful, necessary and important. The benefits may be offset by perceived risks focusing on fear and ethical concerns. Compared to an earlier expert study on societal acceptance of nanotechnology, consumers emphasised ethical issues compared to experts but had less concern regarding potential physical contact with the product and time to market introduction. Consumers envisaged fewer issues with several applications compared to experts, in particular food applications.

## Introduction

Consumer acceptance of different applications of nanotechnology is likely to be a key determinant influencing its future development and implementation trajectory [56]. The potential economic and social benefits of nanotechnology may not be realised if societal responses to its application are not adequately addressed early in the process of application development [43, 49]. However, given the broad range of potential applications, it is unlikely that all applications will be equally acceptable to consumers. Consumer preferences and priorities regarding both the implementation of regulation designed to optimise consumer and environmental protection, and potential characteristics of consumer products, should be given due consideration while formulating regulations, policies and design issues relating to nanotechnology [39, 47], as well as the design of specific applications and products [19, 24]. It has been long established that societal responses to emerging technologies result from a diverse range of considerations, many of which are not technical but instead have their origins in moral, ethical or value-driven concerns [32, 35, 58]. The aim of the current paper is to identify the types of concerns and priorities consumers have about different applications of nanotechnology and to assess the extent to which these align with what various experts involved in the commercialisation of nanotechnology perceive to be the concerns and priorities of consumers. This is important as the commercialisation process will be driven by expert, rather than lay opinions regarding the societal acceptability or otherwise of different applications, while the final success or failure depends on lay responses to the technology.

### Factors Influencing Consumer Acceptance of Nanotechnology

Past research has focused on public attitudes associated with nanotechnology, which has suggested that both risk and benefit perception represents important determinant of nanotechnology acceptance [5, 9, 50, 69]. The results of these studies suggest that, in general, public attitudes towards nanotechnology tend to be reasonably positive and the perceived benefits of nanotechnology tend to outweigh the perceived risks. However, the research focus on *risk-benefit* perception may exclude consideration of broader ethical issues potentially relevant to consumer decision-making [8, 30]. Greater in-depth understanding of potentially influential ethical issues which consumers may, or may not, use in their evaluations of the acceptability or otherwise of nanotechnology and its applications is needed. Previous research [20, 31] has suggested that experts who are involved in the development and regulation of nanotechnology perceive that consumers will accept application which they perceive to be useful and beneficial and reject those which consumers perceive to be risky and to be the potential for misuse. Ethical and moral issues were not reported by experts to be a driver of consumer (non) adoption of specific nanotechnology applications. However, perceived moral concerns held by consumers regarding enabling technologies have been found to be an important determinant of consumer’s acceptance of their applications (e.g. see [25]), and nanotechnology is unlikely to be an exception to this [8, 70]. The objective of the current research was to compare expert and consumer views regarding the factors which potentially influence consumer acceptance of different applications of nanotechnology; using an identical methodology which seeks to eliminate researcher bias is the construction of terminologies. The expert study^1^ has been published elsewhere [31]. The research reported here reports the use of the identical methodology in a consumer sample, enabling a direct comparison of the results to be made. Moral or ethical concerns did not emerge as an important factor determining whether consumers would accept different applications of nanotechnology in the expert study [31]. However, there is evidence to suggest that the extent to which an individual is positive towards science and technology in general is a predictor of their support for nanotechnology [50, 62, 76]. This may, in turn, be influenced by religious beliefs, such that the more religious an individual is, the higher their level of opposition for funding related to nanotechnology development or the lesser their belief is nanotechnology is morally acceptable [4, 61]. Social justice and vulnerability have also been found to influence societal perception of nanotechnology [9]. It has been shown that when the risk-benefit distribution from nanotechnologies is perceived as unfair and/or associated with differential impacts on vulnerable populations, perceptions of risk associated with nanotechnology may increase. This analysis has origins in moral argumentation. The opinions of external information sources and institutions may also influence people’s attitudes towards, and ethical concerns associated with, nanotechnology. The perceived characteristics of different types of nanotechnology application may also influence acceptance. Priest and Greenhalgh [48] reported that most future benefits anticipated by participants in their study were in the areas of medical advances, where benefits are perceived to be more valuable and ethically justified. Conti et al. [9] studied variation in consumer response to energy, food and medical applications of nanotechnology and demonstrated that nano-enabled foods are more likely to raise societal concern than other applications. Perceived “bodily invasiveness” was also reported to be influential in determining acceptance, with most consumer negativity reserved for food-related applications.

### Expert Versus Consumer Perception

The difference between expert and lay assessments of technological, and other, risks has been well established (e.g. see [67]). This corpus of research has suggested that while technical experts make judgements about the severity of the risk based on risk assessments, lay people perceive risks multi-dimensionally (e.g. see [16, 33]). In their daily lives, people make risk assessments based on a number of factors: These include, for example, uncertainty, dread, catastrophic potential, controllability, equity and risk to future generations [68]. This finding is important because, within the psychometric approach, multi-dimensional risk perception is invoked to explain the expert-lay disagreement (but see [55]). From this research, it has been posited that experts fail to understand the psychological factors which determine citizen and/or consumer responses to specific hazards (for example, in relation to consumer goods), which have resulted in poorly thought-through commercialisation policies, in particular in relation to application of technological innovation processes [1, 3, 65, 66, 78]. In particular, the moral or ethical concerns of the public, which are potentially important determinants of consumer response (e.g. see [12, 23, 25, 38, 40]), may have been underestimated by experts with respect to nanotechnology application [8].

Previous research on risk and benefit perceptions associated with new technologies has indicated that understanding and mapping consumer perceptions and attitudes towards emerging technologies, including nanotechnology, can facilitate the “fine tuning” of the technology implementation process to align with consumer preferences and priorities. Even while holding the best intentions to create technologies that benefit society, scientifically trained experts tend to perceive risk differently than the public. Given that scientific and technical experts, as well as experts within the policy community, tend to frame regulatory and implementation policies and commercialisation trajectories for emerging technologies, it is important to both understand consumer perceptions of risks, benefits and moral concerns, and what these are thought to be by expert communities (see also [65, 66]).

### Utilising Methodologies Which Do Not Impose Researcher Biases on Participant Responses

Research into expert and consumer perception and concerns associated with the development and application of new technologies has frequently applied survey methodologies where questions identified as relevant by the researcher were included or where questions were derived from existing theoretical models of technology acceptance. Such an approach may fail to capture the broad range of factors influencing societal response to nanotechnology if these were not considered as important when the study was designed [19, 24, 58]. This is particularly relevant as research regarding consumer perceptions associated with the introduction of emerging technologies must focus specifically on the technology under consideration, as extrapolating from consumer responses to other technologies previously introduced may not necessarily be appropriate [22]. An important element in identifying citizen concerns and innovation priorities is to allow them to express these in their own words and develop predictive “constructs” which can be used to differentiate different applications of nanotechnology in a way which is meaningful to study participants [10, 11, 31, 46, 63].

### Repertory Grid Method

An established methodological approach that is well suited to eliciting perceptions and values without framing these as part of the research process is that of repertory grid methodology [37], applied in conjunction with generalised Procrustes analysis (GPA) [27]. The repertory grid method (RGM) originated in psychology and has been used in number of studies across different disciplines to elicit individual’s perception on new technologies and/or products [21, 22, 31, 41, 44, 45, 54, 57, 71]. The repertory grid method was not designed as a methodology for public participation, which typically depends on the interaction between participants to gain increased insights into the issue at hand (e.g. see, inter alia, [53]). However, information about public perceptions can be derived from the application of repertory grid methodology, which can be addressed in policy decision-making processes, and these are reviewed elsewhere (e.g. [52]). These include, for example, deliberative conferences and citizen’s juries (see, [53]). However, these methods, while allowing for deliberation on the part of participants, also rely on the provision of extensive amounts of information, to which the general public in many real-life cases will not have access. To elicit in-depth understanding of information about consumers opinion on different applications of nanotechnology, focus groups and interviews may be applied [75]. Typical of focus groups is that they include social interaction between participants and thus is a reflection of real-life social opinion formation. On the other hand, because of the social interaction, focus groups may result in consensus within the groups which will overwhelm individual (minority) opinions. Interviews are particularly useful to deliver in-depth insight into specific motivations and thought of individuals on a limited set of topics [17]. Repertory grid interviews are a specific type of interview that aims to identify common evaluative dimensions across a range of stimuli. In the repertory grid method, a structured interview format is adopted, which limits the amount of interpretative data gathered beyond the predefined interview structure [18]. Repertory grid can be used as a research tool to facilitate a stakeholder dialogue on a societal issue [74] and is particularly valuable in consumer research in the early stages of product development [75]. Repertory grid methodology provides a structured methodology in which individual perceptions of the issue of interest can be explored without extensive imposition of researcher bias or vocabulary on participant responses, although it is arguable that methodological biases cannot be eliminated completely [45, 60]. The method is efficient in identifying the full range of constructs that people use for evaluating an issue in a particular context with as few as 15 interviews [74].

The data from repertory grid method can be analysed using GPA. This allows the identification of constructs about which respondents agree, so that the most important determinants of acceptance or rejection can be identified. GPA is a multivariate statistical technique that aims to identify consensus between respondent assessment patterns and provide a measure of respondent agreement with as little intervention on the part of the researcher as possible [79]. It is particularly useful when information is required about how individuals differ and to what extent they agree in their perception of the same topic (in this case, specific applications of nanotechnology) [13] and where the researcher is interested in excluding researcher bias from the results. By analysing the results using GPA, variations due to respondents using different terms to describe the same stimuli and/or variation in their use of rating scales can be controlled [45].

The current research therefore aims to elicit the factors that shape consumer perception of different applications of nanotechnology using the same methodology as previously applied to experts and compare consumer views elicited in this study with the views of experts^2^ regarding consumer perceptions which have previously evaluated, [31] through application of repertory grid and generalised Procrustes analysis.

## Methods

### Participants

Structured interviews were carried out with 18 participants, 10 men and 8 women (mean age = 40.6 years, SD = ±14.5 years), recruited from Newcastle Upon Tyne in the UK by a social research company, and selected from a range of ages and socio-economic groups. Although a sample of this size cannot be said to be nationally representative, the respondents represented a good cross section of UK age and socio-economic groupings.

### Design

In order to identify the factors which consumers perceive will drive societal acceptance of different applications of nanotechnology, respondent opinions linked to different nanotechnology products were elicited. The repertory grid method was utilised. Respondents were asked about 15 different applications of nanotechnology drawn from different areas of application (e.g. medicine, agriculture and environment, chemical, food, military, sports, and cosmetics). Respondents were asked about 15 applications of nanotechnology drawn from different areas (e.g. medicine, agriculture and environment, chemical, food, military, sports, and cosmetics). For both nanotechnology in general, and the 15 applications under consideration, a brief explanation was provided (the full text is provided in Table 1). The survey used 10 triads (each triad consisted of set of 3 applications) compiled from the 15 specific applications of nanotechnology to initiate construct elicitation. Triads were presented in randomised order with each application being presented twice (in different triads) to each respondent. For each triad, respondents were asked. For each triad of nanotechnology applications, respondents were asked, “which 2 out of these applications of nanotechnology do you find to be similar in terms of societal response, and why?” They were asked to identify which two applications they thought would be most likely to evoke similar societal responses and to explain their decision. They were also asked to explain why the application which they thought would evoke a different societal response would be viewed differently by the public through use of the question “which of these applications of nanotechnology is different from the other two applications in terms of societal response, and why?” The answers were used to create bipolar arguments on differences between the applications. Once all 10 triads had been used to elicit arguments, respondents evaluated each application of nanotechnology, against each bipolar construct developed from their repertory grid interview on a 5-point scale, with one pole of the construct at score 1 and the other pole at score 5.

**Table 1:** Descriptions of nanotechnology and three agri-food applications of nanotechnology

Nanotechnology	Nanotechnology is a technology that is based on the use of very small particles (nanoparticles). Nanoparticles are smaller than 100 nm. For comparison, a human hair is, on average, about 60,000 nm thick (in other words, nanoparticles are very small indeed). One nanometre is only one billionth of a metre in size. Nanoparticles can be used to design and deliver new applications of products and services, for example in medicine, cosmetics, material engineering and food products.
Smart pesticides	Smart pesticides are new generation of chemicals used for crop protection in agriculture. Pesticides are encapsulated using nanoparticles so to minimise the doses of pesticide which are used on the crop and to get maximum effect with more targeted action of the pesticides.
Encapsulation and delivery of nutrients in food	Nanoparticles are used to “encapsulate” or enclose vitamins or other nutrients and carry them through the stomach straight into the intestine to be absorbed by the bloodstream. This means that they are not broken down in the stomach and so can be used by the body to improve health.
Food packaging	Nanotechnology can be applied to develop synthetic food packaging that kills germs which cause foods to “go-off”. This means that the food can be preserved for longer periods of time. The packaging is described as having “antimicrobial “properties.

### Procedure and Data Collection

The data were collected in a face-to-face interview. All respondents were given a short description of nanotechnology at the outset of the experiment. The interview was divided into two phases. In the first phase, constructs describing determinants of societal response to nanotechnology were elicited. This was followed up during the second phase where the respondents rated each of the applications on each construct they had personally described as relevant. The interviews were conducted between March 13 and 23, 2012, using Idiogrid software [28]. All interviews were audio-taped and transcribed after receiving verbal consent from the interviewee. On average, it took 55 min to complete the interview.

### Analysis

The aggregated data from the 18 participants consisted of 360 constructs in total. The number of constructs elicited from each participant ranged from 19 to 20, with the mean number of constructs being 19.6. The first author classified the constructs into series of construct classes. Subsequently, the second author applied the initially defined construct-classes to the constructs. A Cohen’s kappa of 0.74 indicated good agreement between the coders regarding the classification of the constructs. Differences were then resolved by discussion to achieve consensus on classification, and in total, 60 construct classes were finalised (Appendix). The construct classes were based on abstractions of the actual constructs; for example, if a respondent stated that he or she found the applications “useful for particular section of society”, this was deemed to fall within the class of “useful for subgroup of people”. Some constructs were classified as combination of two construct classes for e.g. “health benefits + environment benefits”, in which case they were considered to contribute 0.5 to each class.

The grid data for each respondent derived from repertory grid interviews was submitted to GPA using Idiogrid software. A GPA group average perceptual space was obtained, illustrating the relative positions of the 15 applications of nanotechnology. GPA allows each respondent to have unique set of attributes by transforming the resulting data by translation, rotation or reflection in order to find consensus among the respondents. The consensus proportion obtained using GPA represents the average of all the transformed configurations. Interpretation of this consensus helps in the identification of the most salient constructs. To interpret the dimensions of the profile spaces derived from GPA, principal component analysis (PCA) is performed using Promax rotation, on the consensus grid obtained from GPA [29]. To identify the most salient psychological constructs, the structure loadings for each construct were examined on the principal axis. To select the principle construct classes, a pragmatic approach has been adopted where only those construct classes occurring three or more times with loading coefficients (≤−0.50 or ≥0.50) were considered to be important. These were further used for labelling the principal component on which it loaded (bold, Table 2). Once the most salient constructs were identified using PCA, the transcribed interviews were reviewed to identify statements illustrative to the specific consumer view captured by the constructs and explained differences in consumer perception associated with the different applications of nanotechnology.

**Table 2:** Comparison of nanotechnology applications on different factors: means (standard errors) based on repeated measure ANOVA

Factors^a^	Smart pesticide	Nano-encapsulated food	Food packaging	Importance of factor for introduction of nanotechnology (1 = extremely important, 5 = not at all important)^b^
Concerns about technological implementation (1 = extremely likely to raise concern, 5 = not at all likely to raise concern)^1^	2.80 (.05) a	2.78 (.05) a	2.95 (.05) b	2.26 (.05) I
Benefits and need (1 = extremely beneficial and necessary, 5 = not at all beneficial and necessary)^1^	3.16 (.06) a	3.01 (.06) b	2.97 (.06) b	2.60 (.05) II
Access to technology (1 = extremely likely to have access, 5 = not at all likely to have access)^1^	3.30 (.06) a	3.37 (.07) a	2.98 (.07) b	2.81 (.06) III
Risk and fear (1 = extremely risky and frightening, 5 = not at all risky and frightening)^1^	3.57 (.05) a	3.71 (.05) b	3.79 (.06) b	2.27 (.05) I

*N* = 417

^a^Means with different letters are significantly different between applications or factors (pairwise comparisons LSD; *α* = 0.05)

^b^Means with a different superscript Roman numeral are significantly different between applications or factors (pairwise comparisons LSD; *α* = 0.05)

## Results and Interpretation

The consensus proportion was 0.62 indicating that the GPA consensus grid represented participant views about the 15 applications with respect to their self-generated constructs reasonably well. The consensus proportion was tested for statistical significance using a randomisation test [77]. Simulations of 1000 trials were generated based on the current data and showed that the observed consensus proportion was not the result of a random dataset (significant at *p* < .001). The consensus proportion for the two most extreme respondents was 0.25 and 0.67, respectively, indicating that there was relatively little variance in response with respect to the consensus grid. Consensus ratio’s for applications of nanotechnology ranged from 0.33 to 0.75 (Table 3). Higher consensus was found for applications such as easy to clean surfaces, smart dust, environment remediation, sports goods, water filtration and medical applications of nanotechnology. More variation between participant opinions was found for applications such as nano-fabrics, encapsulation of nutrients in food, food packaging, chemical sensors, fuel cells and cosmetics.

**Table 3:** Estimated marginal mean scores (standard errors) of trust in information based on repeated measures ANOVA

Institutions	Trust in information (1 = no trust at all, 5 = extremely high trust)^a^
Universities	3.29 (.05) a
Industry	2.51 (.05) b
Government/ Regulatory agencies	2.61 (.05) b
Consumer organisations/ Public NGOs	3.03 (.05) c
Environmental NGOs	3.01 (.05) c
Patient groups	3.04 (.05) c
Media	1.97 (.05) d
Insurance companies	2.01 (.05) d

^a^Means with different letters are significantly different between applications or factors (pairwise comparisons LSD; *α* = 0.05)

Interpretation of the results was limited to first three principal components (PC), based on the criterion eigenvalues > 1 (with the first four eigenvalues being 4.16, 2.21, 1.07 and 0.75). The three components together accounted for 74.3 % of the variance. Table 2 lists the number of construct classes that have high correlations with the first three PC. Only those construct classes that occur three or more times are considered important for interpretation. A number of construct classes were identified that loaded on more than one principal component. This can be interpreted by taking into account the correlation between the components allowed for in the Promax procedure. That is, if two components are correlated then a construct class related to one of the components will to some extent be also related to the correlated component. Moderate correlation [7] was found between PC1 and PC2 (0.35) and between PC2 and PC3 (0.32). A negligible correlation [7] of 0.05 was found between PC1 and PC3.

**Fig. 1:**
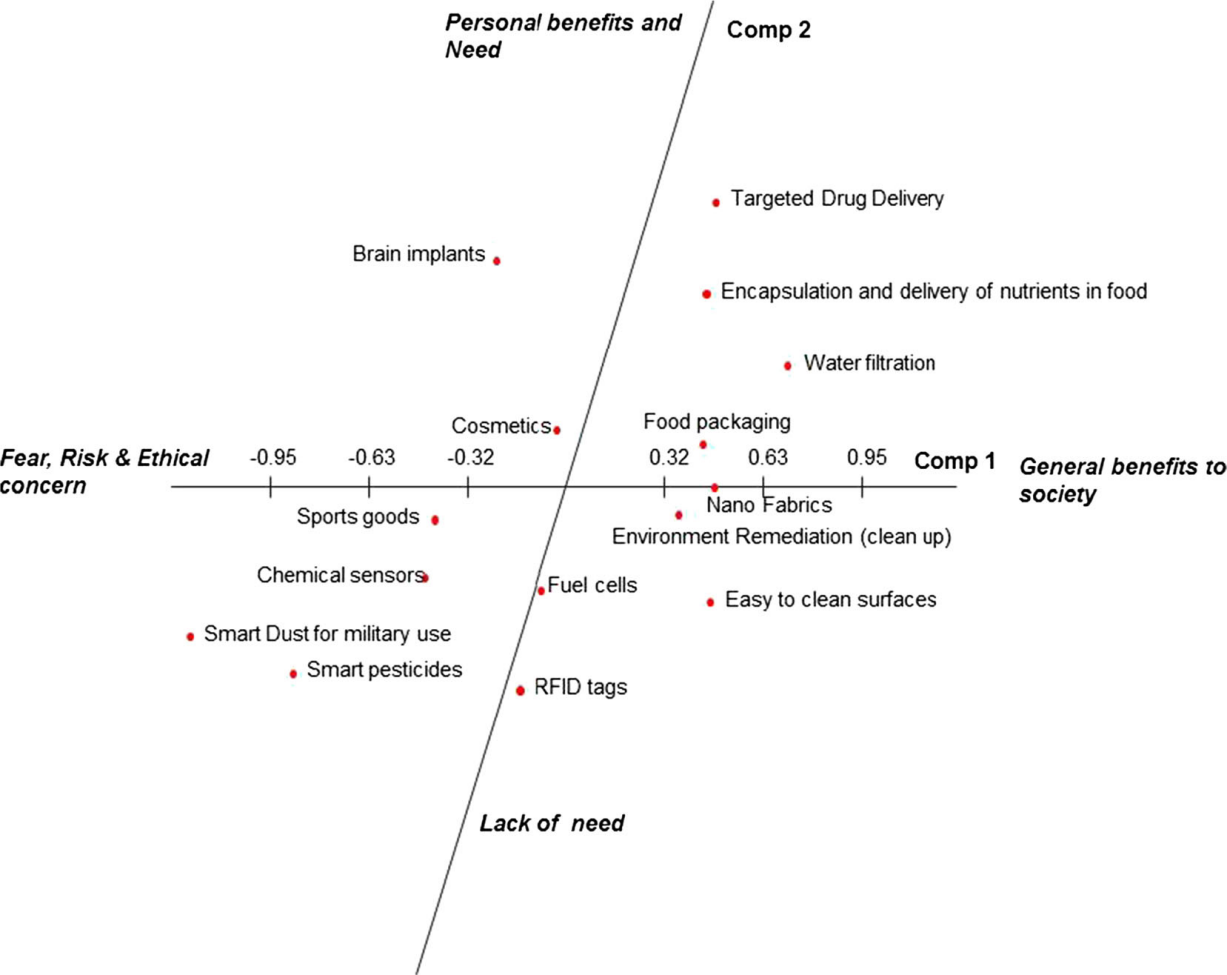
Location of applications of nanotechnology on first and second principal component

**Fig. 2:**
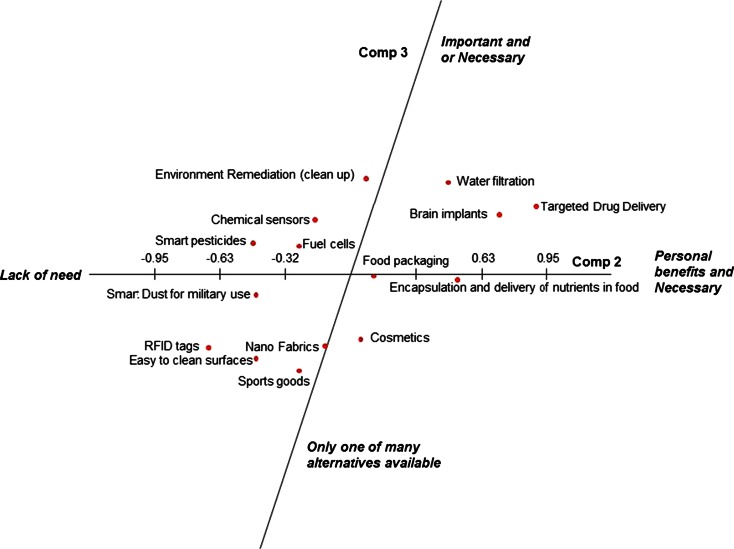
Location of applications of nanotechnology on second and third principal component

The first principal component (PC1) explains most of the variation in the data. The constructs used to describe determinants of consumer response to different applications of nanotechnology combined towards the positive end of PC1 are “beneficial for more people”, “general benefits”, “daily use”, “desired by everyone”, “environmental benefits”, “health benefits”, “makes lifestyle easy”, “necessary” and “useful for general public”. The positive end of PC1 was interpreted as “general benefits to society”. The negative pole of PC1 was described by the constructs “could be misused\abused”, “doubts”, “fear”, “generally fewer or no benefits”, “no knowledge”, “not acceptable to society”, “less\not desirable”, “privacy concern” and “useful for subgroup of people” (Fig. 1), reflecting negative attitudes. This component was therefore labelled as “fear, risk and ethical concern”. Constructs such as “daily use”, “makes lifestyle easy”, “could be misused\abused”, “fear”, “no personal knowledge”, “not acceptable to society” and “privacy concern” were found to only load on PC1. PC2 explains second greatest proportion of the variation in the data. The positive pole of PC2 relates to constructs such as “desired by everyone”, “health benefits”, “necessary”, “personal benefits”, and “useful for general public”. Therefore, this pole of PC2 can be described as “personal benefits and need”. Constructs found on the negative pole of PC2 are “environmental benefits”, “generally fewer or no benefits”, “less\not necessary”, “less\not important”, “less\not desirable” and “useful for subgroup of people” and can be characterised as “lack of need” (Fig. 1). The third principal component (PC3) has its positive pole associated with “beneficial for more people”, “general benefits”, “environmental benefits”, “health benefits”, “important”, “necessary” and “useful for subgroup of people”. This pole of PC3 can be labelled as “important and/or necessary”. Constructs highlighted on the negative end of PC3 are “alternatives are available”, “doubts”, “less\not necessary”, “less\not important”, “nice to have” and “personal benefits”, and therefore the negative side of PC3 can be defined as “only one of many alternatives available ” (Fig. 2). Constructs found to only load on PC3 are “important”, “alternatives are available” and “nice to have but not essential”.

On the basis of the constructs associated with each principal component, it is possible to make some inferences about how respondents have characterised the 15 applications of nanotechnology. Along PC1 (Fig. 1), which is primarily associated with general benefits to society at one pole along a continuum with risk, fear and ethical concerns associated with the other, applications such as *water filtration*, *easy to clean surfaces*, *nanofabrics*, *encapsulation and delivery of nutrients in food*, *food packaging*, *targeted drug delivery and environmental remediation* are positioned positively, indicating these applications to be associated with general benefits to the society and health and environment benefits:

“Water filtration will lead to massive humanitarian benefits to society” (Respondent 16)

“Most people will be happy to receive nanomedicine” (Respondent 2)

“Nanofabrics may lead to use of less detergent hence an environment friendly product” (Respondent 11)

“Environmental remediation is completely beneficial as it leads to sustainability” (Respondent 13)

Applications such as *RFID tags*, *chemical sensors*, *brain implants*, *sports goods*, *smart pesticides* and *smart dust for military use* were rated less positively on this continuum, indicating these applications to be rated by respondents as being risky, invoking fear, raising ethical concerns such as concern regarding equity of distribution of benefits, invasion of privacy and doubts whether the technology will be able to deliver the benefits it promises:

“RFID could put people out of jobs” (Respondent 1 and 14)

“RFID tags would be invasive, and people are always defensive about such applications” (Respondent 16)“Nano pesticide need to be tested fully to confirm that there is no leakage from one field to another” (Respondent 2)

“I would be very uneasy with brain implants as brains are very complicated, same with smart dust, doubts on what kind of information others might collect” (Respondent 8)

*Cosmetics* and *fuel cells* were rated as neutral along this continuum, indicating that participants consider these to neither hold much benefits nor be unacceptable based on overall acceptability to society. Of all the applications, *smart dust* was seen as most risky, while *water filtration* was seen as the most beneficial application of nanotechnology to society.

PC2 (Fig. 1) differentiates different applications on the basis of how necessary they are perceived to be. *Targeted drug delivery*, *brain implants*, *water filtration*, *encapsulation and delivery of nutrients in food*, *food packaging*, *environmental remediation and cosmetics* are positioned on the positive side of this continuum, indicating that they are associated with personal benefits and are deemed more necessary, for example,

“Brain implants and water filtration could be necessary for a lot of people” (Respondent 10)

“Nanofood, I would buy it personally, as it will be a positive effect to have added nutrients in your food” (Respondent 10)

“Cosmetics would give more personal benefits” (Respondent 17)

On the negative side of PC2 are applications such as *nanofabrics*, *chemical sensors*, *fuel cells*, *sports goods*, *RFID tags*, *easy to clean surfaces*, *smart pesticides* and *smart dust for military use*. Lack of need was seen to be associated with these applications of nanotechnology:

“Easy to clean surfaces is nice to have but not really necessary” (Respondent 10)

“Smart pesticides are not necessary” (Respondent 12)

PC3 (Fig. 2) corresponds to the distinction between applications that are considered important and or necessary to applications that are considered to be only one of the many alternatives available to the public. Applications located on the positive side of PC3 are *water filtration*, *environmental remediation*, *targeted drug delivery*, *brain implants*, *chemical sensors*, *fuel cells* and *smart pesticides*. These applications are considered as being more important and/or necessary:

“Brain implants are really important…if you have illness” (Respondent 1)

“Medical applications are definitely most important ones” (Respondent 4, 12)

“Water filtration will be very important for the third world countries” (Respondent 8, 11)

On the negative side of this dimension were applications such as *smart dust for military use*, *nanofabrics*, *cosmetics*, *RFID tags*, *easy to clean surfaces* and *sports goods*, which are described as only one of many alternatives.

“Fabrics you can have alternatives” (Respondent 9)

“Easy to clean surfaces is no added value using nanotechnology, we can do the cleaning ourselves” (Respondent 12)

*Food packaging* and *encapsulation and delivery of nutrients in food* were rated as neutral applications on this continuum on PC3.

Targeted drug delivery and water filtration are applications which the participants in the study consider promising on all three dimensions (in top right section in both Figs. 1 and 2). Smart dust for military use and application of nanotechnology to produce sports goods appears to be received negatively (lower left section in both figures). For the other applications considered, participants expressed a more neutral view (Table 4). For example, nano-fabrics are considered positive for society as whole because of their potentially positive impacts on the environment (reduced use of detergent). Participants did not perceive personal benefits to be associated with nano-fabrics (PC2), and participants expressed the view that alternative approaches to reaching the same goals may also be developed (PC3). From here, it is predictable that people may not object to nano-fabrics, as the societal benefits are appreciated.

**Table 4:** Participant segmentation based on their views on different factors (*N* = 292)

		Cluster 1 *Moderate*/*neutral*	Cluster 2 *Risk averse*	Cluster 3 *Benefit seekers*	Repeated measures ANOVA
Number		145	60	87	
Clustering variables					
Risk-fear	Smart pesticides	3.9	2.8	3.4	Interaction effect: *F* (3.88, 560.62) = 1.27; *p* = 0.28
	Nano-encapsulated food	4.1	2.9	3.4	
	Food packaging	4.2	2.9	3.4	
	Overall	4.1	2.9	3.4	Main effect: *F* (1.94, 560.62) = 5.16; *p* < .01
Importance: risk-fear		2.7	1.7	1.9	*F* (2, 289) = 36.42; *p* < .001
Benefit-need	Smart pesticides	3.2	4.1	2.2	Interaction effect: *F* (3.86, 558) = 3.78; *p* < .01
	Nano-encapsulated food	2.9	4.1	2.1	
	Food packaging	2.8	4.3	2.2	
	Overall	3.0	4.1	2.1	Main effect: *F* (1.93, 558) = 2.16; *p* = 0.12
Importance: benefits-need		2.6	3.2	1.9	*F* (2, 289) = 51.8; *p* < .001
Concerns about technological implementation	Smart pesticides	3.1	2.1	2.6	Interaction effect: *F* (3.91, 564.27) = 3.98; *p* < .01
	Nano-encapsulated food	3.2	2.1	2.6	
	Food packaging	3.5	2	2.6	
	Overall	3.3	2.09	2.6	Main effect: *F* (1.95, 564.27) = 1.81; *p* = 0.30
Importance: concerns about technological implementation		2.7	1.7	1.9	*F* (2, 289) = 40.26; *p* < .001
Accessibility to technology	Smart pesticides	3.7	3.4	2.5	Interaction effect: *F* (4, 567.18) = 0.59; *p* = 0.67
	Nano-encapsulated food	3.8	3.5	2.6	
	Food packaging	3.4	3	2.3	
	Overall	3.6	3.3	2.4	Main effect: *F* (1.96, 567.18) = 16.62; *p* < .001
Importance: accessibility to technology		2.8	3.5	1.9	*F* (2, 289) = 49.09; *p* < .001
Interpretation variables					
Gender (% female)		53 %	58 %	44 %	*X* ^2^ (2, *N* = 292) = 3.4, *p* = .18
Age	18-25	14.5 %	10.0 %	20.7 %	*X* ^2^ (8, *N* = 292) = 19.07, *p* < .05
	26-35	20.0 %	11.7 %	28.7 %	
	35-54	46.9 %	51.7 %	29.9 %	
	55-65	15.9 %	23.3 %	12.6 %	
	65+	2.8 %	3.3 %	8.0 %	
Education level	No formal qualification	6.9 %	8.3 %	12.6 %	*X* ^2^ (10, *N* = 292) = 11.07, *p* = .35
	Vocational qualification	6.2 %	3.3 %	11.5 %	
	GCSE/O level	40.0 %	38.3 %	32.2 %	
	A level	24.1 %	23.3 %	13.8 %	
	Bachelor’s degree	13.8 %	18.3 %	19.5 %	
	Post-graduate degree	9.0 %	8.3 %	10.3 %	
Familiarity at all (% yes)		30 %	43 %	39 %	*X* ^2^(2, *N* = 292) = 4.26, *p* = .12
Knowledge		(*N* = 43) 3.7	(*N* = 26) 3.6	(*N* = 34) 2.6	*F* (2, 100) = 18.68; *p* < .001
Trust		2.73	2.50	3.03	*F* (4.87, 1408.45) = 135.89; *p* < .001

## Discussion

Consistent with findings from previous studies [14, 46, 51, 59, 76], the majority of participants reported being unfamiliar with nanotechnology and its various applications. Despite this, participants were able to differentiate between different applications of nanotechnology on the basis of perceived need, benefit, usefulness and importance. Environmental and health risk, fear and ethical concerns were also considered to be factors potentially influencing acceptance.

The results suggest that people may differentiate applications of nanotechnology based on their perceptions of associated benefits. These perceived benefits may also vary according to the type of application, for example, health benefits were associated with food and medical applications of nanotechnology, while environmental benefits were linked to fuel cells, environmental remediation and nano-fabrics. In addition, applications were also differentiated on the basis of whether or not they bring benefits to society as a whole or whether these benefits are experienced by individuals. Overall, water filtration was rated as the most beneficial application of nanotechnology. Even though the majority of the participants felt that they would not need this application in the UK, they simultaneously acknowledged that water filtration using nanotechnology will bring benefits to developing countries. Medical and environmental applications were seen as the most beneficial applications, in line with existing research [9, 48]. Food applications were also rated as beneficial for society and individuals, in contrast to previous studies [6, 10, 63, 64, 76]. This may be attributable to methodological differences raising benefits as primary and risk and other concerns as secondary topic, where most other studies have utilised researcher-generated constructs that often include risk perception as a central topic, making risk more central in the mind.

The concepts of need, usefulness and importance emerged as important constructs. According to participants, the perceived benefits alone would not be the decisive factor in societal acceptance, but other relevant factors, such as the extent to which the application is perceived to be important or necessary, or “trivial”, will shape consumer responses to different applications. Food and medical applications were seen to be the most useful and necessary applications of nanotechnology, while applications such as RFID tags, and smart dust for military use, were viewed as unnecessary. Nanoproducts such as sports goods, cosmetics and nano-fabrics were seen as applications where different technological approaches would deliver products with the same properties, and so the use of nanotechnology in their production was considered unwarranted.

The results also suggest that consumer and/or citizen will be driven by perceptions of fear, ethical concerns and low levels of (perceived) knowledge about the different applications of nanotechnology. In this study, applications such as RFID tags were associated with negative socio-economic impacts (such as unemployment), suggesting that these may be as relevant as health and environmental risk in determining acceptance. Doubt and uncertainty regarding the equity of the distribution of the benefits emerged as an important ethical concern among the participants. For applications such as brain implants, pesticides, smart dust and cosmetics, participants doubted that everyone would have access to the benefits associated with such applications, suggesting that the ethical principle of equity of distribution of benefit may be compromised. In addition, there were doubts about whether certain applications would deliver the benefits as promised or would be oversold in terms of the benefits they will potentially deliver. Participants also expressed that they feared misuse of applications such as smart dust, RFID tags and pesticides. The first two of these applications were seen to raise privacy concerns.

Some limitations of the research merit discussion. Although these outcomes resulted in a robust factor structure following statistical analyses, it is conceivable that the outcomes are, in part, influenced by the selection of nanotechnology applications utilised in the study design and to which participants had to respond. These applications were carefully selected to cover a broad range of potential uses of nanotechnology. In practice, however, the more easily manufactured and/or less controversial applications may be, the most likely to be commercialised. The selection of applications used in this study may have included a relatively high proportion of potentially controversial products, which may have influenced the results. However, there is some face validity associated with the results, insomuch as they broadly align with other studies on societal the acceptance of different applications of nanotechnology (for example, [63]).

The lack of knowledge of consumers about nanotechnology may also have influenced the results. If members of the public lack knowledge, they may utilise what knowledge they do have and disregard the information they do not understand [2]. In the research reported here, participants may have evaluated the utility of the different applications, instead of the use of nanotechnology in their development. Nevertheless, the outcomes of the research remain relevant. Even for an area of application which is potentially societally controversial (for example, the development of novel pesticides), it is likely that the controversial properties of the application will become associated nanotechnology in the mind of consumers. This emphasises the need to introduce nanotechnology in the context of applications and products which are attractive to the consumer or end-user.

The use of the repertory grid method allowed identification of elements often overlooked in similar research, such as the perceived (personal) need for a specific nanotechnology application (dimension 2 in the generalised Procrustes analysis). Another insight identified from the application of repertory grid methodology, when compared to studies adopting predefined questions, is that, in the current study, potential benefits associated with the different applications of nanotechnology were consistently mentioned prior to potential risks. The application of the repertory grid method suggests that when people are thinking freely, perceived benefits are the first criterion on which different applications are compared.

How does this relate to the way experts thought that the consumer would perceive the same applications? Conducting such a comparison facilitates understanding how consumer and expert views align or misalign. Knowing where potential alignment and misalignment occurs can not only support effective communication about nanotechnology but can also be used to inform the design of specific applications of nanotechnology to facilitate societal relevance and acceptability. Such a comparison is enabled by the methodological similarities between Gupta et al. [31] to assess expert views of the factors driving consumer acceptance and the actual views held by consumers. Both experts and consumers identified the main factors influencing societal response to different applications of nanotechnology as being the extent to which applications are perceived to be *beneficial*, *useful* and *necessary*. Two additional factors were deemed important for consumer acceptance by experts that were not raised by the consumer sample. These were concern *over end-users coming into contact with the nanomaterials used* and *how close to the market is the application*. In contrast, consumer participants identified one issue that was *not* identified by experts, related to *ethical concerns*, and the issue of *equity of distribution of the benefits as* potentially influential determinants. Future research might usefully be to explore the specific role of ethical values in shaping consumer perception of nanotechnology and to systematically relate ethical concern to other concerns such as risk and safety.

It is postulated that the expert focus on technical issues being the primary determinant of consumer responses to different applications of nanotechnology may be attributable to their own concerns about technical issues associated with developing beneficial and safe products, which are translated into societal priorities for implementation. For example, experts suggest that the consumers will be concerned about physical contact with nanomaterials. This may be attributed to expert knowledge about exposure to hazardous materials. One interpretation is that, when experts are aware of the importance of societal acceptance and try to identify potential drivers of societal response, they nonetheless focus more on the technical issues associated with different applications of nanotechnology. Against this, consumers are also concerned by ethical and moral issues, as indicated by differentiation on the risk, fear and ethical concern dimension which emerged from the analysis. This would include, *inter alia*, issues relating to the equity of distribution of benefits associated with specific nanotechnology applications, so as not to disadvantage specific groups in the population and concerns about introducing *unnecessary* novel risks if the same advantages can be obtained through application of conventional means. Such moral and ethical issues were not identified as relevant by experts but may ultimately lead to rejection of the technology and its (specific) applications by consumers.

Both experts and consumers indicated that medical applications and water filtration are likely to be considered as the most beneficial and necessary applications of nanotechnology. However, whereas experts perceived that food-related applications of nanotechnology (smart pesticides, encapsulation and delivery of nutrients in food and packaging) will be less acceptable to society, the consumers included in this study perceived food applications of nanotechnology to be beneficial, suggesting that experts may be overly concerned about food applications. One interpretation is that experts impute the negative consumer reactions to GM foods (in particular in Europe as the “normative” consumer response to all emerging technologies applied to food production (see *inter alia*, [19, 24, 72, 73]). Extrapolation of consumer concerns from the GM foods example may not reflect socio-political changes which have occurred since their introduction (for example, the enactment of increased regulation designed to promote consumer protection). In addition, changes may have been made in technology development trajectories such that applications with obvious consumer benefits are prioritised in terms of market entry. Furthermore, socio-economic contexts may have changed (for example, lower prices may be more of a priority in a recession when consumers have lower disposable incomes). There has been substantially less societal discourse associated with the development and application of nanotechnology compared to GM foods. Taken together, it may be that the view promulgated by some experts that the societal response to GM food technology in Europe is the normative societal response to technological innovation in the agrifood sector is inaccurate. However, the role of ethical and moral concern in determining consumer acceptance should not be underrated by expert communities.

Developing dialogue between technologists and the consumers will ensure that social and ethical issues are addressed early in the development process of emerging technologies. As long as consumer attitudes towards nanotechnology remain largely uncrystallised [15], constant re-evaluation of what the consumer thinks” is required, as these attitudes are unlikely to be static but rather influenced by external events, including the order of entry of products into the marketplace. Such research might draw on both public engagement with smaller groups of people and large representative surveys [36, 42]. Findings from these studies can further add to the ongoing nano debates and governance (see [8, 20] for ongoing nano-governance discussion).

Additional studies are required in different countries and socio-cultural contexts, not least because of the small and unrepresentative size of the sample studied [4, 25, 26, 34]. The results obtained from a sample in one country may not be applicable to populations in another. Nonetheless, this study has allowed comparison of expert and consumer views of what will determine the societal acceptance or rejection of different applications of nanotechnology. The present study shows that even in the absence of risk information related to nanotechnology applications, consumers do spontaneously take moral, ethical and social risk aspects into consideration while discussing acceptability or rejection of nanotechnology applications or rejection of nanotechnology applications, which had not been identified as an issue by experts.

## Footnotes

1 The expert sample was drawn from north-west Europe that comprised of experts (both scientific experts and opinion leaders) from academia, industry, government, media and consumer representative groups who were engaged in diverse activities related to nanotechnology.

2 The experts received no additional information and were not informed that the consumer study was being conducted subsequent to the expert study. Indeed, the consumer study had not been planned at the time that the expert study was conducted.
